# Discontinuation of etanercept after clinical remission in patients with juvenile idiopathic arthritis

**DOI:** 10.1186/1546-0096-10-S1-A51

**Published:** 2012-07-13

**Authors:** Jean-David Cohen, Dominique Fournet, Catherine Ludwig, Marie-Cécile Bozonnat, Michel Rodiere, Christian Jorgensen

**Affiliations:** 1 CHU Arnaud de Villeneuve, Montpellier, France; 2 CHU Lapeyronie, Montpellier, France; 3 Institut Universitaire de Recherche Clinique, Montpellier, France

## Purpose

To evaluate the disease course of patients with Juvenile Idiopathic Arthritis (JIA) after discontinuation of etanercept (ETA) and to determine prognostic factors of clinical remission off medication.

## Methods

Among patients with JIA treated with ETA from 2000 (n = 46), we identified patients in remission defined by a sustained good clinical response according to physicians of our departments for at least 1,5 years and discontinued ETA. The disease course was evaluated using physician and parent/patients VAS for disease activity, active joints, joints with LOM, CHAQ, ESR, CRP. A bivariate analysis of the relationship between JIA subtype, sex, age at onset JIA, disease duration at start of ETA, MTX association, time on ETA (before tapering and total), clinical remission on ETA (defined by Wallace) and disease course was undertaken using the Chi-square or Fisher’s exact test and the Wilcoxon test.

## Results

Among the 25 patients in clinical remission on medication, 16 have discontinued ETA at the moment of the analysis. Main characteristics were: 12 (75 %) female, mean age at onset JIA was 5,5 years (1,4-13,2), JIA subtype were oligoarthritis 7 (44%), polyarthritis 1 (6%), systemic arthritis 5 (31%), enthesitis related arthritis 2 (12,5%), mean disease duration at start of ETA was 3,3 years (0,2-13), MTX was associated to ETA in 11 (69%) cases, median total time on ETA was 2,15 years (1,7-3,2). Discontinuation of ETA was progressive for all patients except 3. After discontinuation, 13 patients are in clinical remission with a median period of 1,8 years (0,5-2,2). Follow-up mean period (after ETA withdrawal) for these patients was 2,45 years (0,5-5,4). Only 3 patients experience flare with a good response after reintroduction of ETA. Median time to flare was 1,7 years (0,4-1,8). The bivariate analysis didn’t reveal baseline values able to predict successful discontinuation of ETA without flare.

## Conclusion

Our retrospective study confirms the possibility of ETA discontinuation in JIA in case of long term of remission. We did not identify predictive factors probably because of the small cohort and, moreover, low number of patients experiencing flare.

## Disclosure

Jean-David Cohen: None; Dominique Fournet: None; catherine Ludwig: None; Marie-Cécile Bozonnat: None; Michel Rodiere: None; Christian Jorgensen: None.

